# Copyrolysis of Municipal Sewage Sludge with Agricultural Residues: A Theoretical and Experimental Study for Tailored Biochar Production

**DOI:** 10.1021/acsomega.4c11089

**Published:** 2025-05-19

**Authors:** Naeimeh Vali, Samyar Zabihi, Abas Mohsenzadeh, Anita Pettersson

**Affiliations:** Swedish Centre for Resource Recovery, Faculty of Textiles, Engineering and Business, University of Borås, 501 90 Borås, Sweden

## Abstract

Municipal sewage sludge (MSS) has been identified as a promising feedstock for producing biochar with potential applications as a soil conditioner and animal feed. However, the high heavy metal content and limited availability of nutrients, such as phosphorus (P), pose significant challenges. This study aimed to improve the quality of MSS-derived biochar through copyrolysis with wheat straw (rich in K and Si) and bakery waste husks (rich in K) at temperatures of 500, 650, and 900 °C. Thermodynamic equilibrium calculations (TEC) were performed using FactSage and HSC Chemistry to predict the stability of P-bearing compounds and the fate of heavy metals in the biochars. The morphology and physicochemical properties of the biochars were examined by using SEM and Brunauer–Emmett–Teller (BET) analyses. The results indicate that increasing the proportions of wheat straw and bakery waste husks, along with higher pyrolysis temperatures, reduced the biochar yield. TEC demonstrated that these blends enhanced the formation of plant-available phosphates compared with pure MSS biochar. This improvement was primarily because of the formation of K/Mg-bearing phosphates in different amorphous and crystalline phases, such as K_2_P_2_O_7_, CaK_2_P_2_O_7_, KPMgO_4_, and KZnPO_4_, instead of Fe/Al-based phosphates. Additionally, copyrolysis reduced the concentrations of heavy metals such as cadmium (Cd), lead (Pb), and zinc (Zn) in the biochars compared to MSS pyrolysis alone. However, it had no significant effect on the copper (Cu), chromium (Cr), and nickel (Ni) levels. In conclusion, copyrolysis with wheat straw and bakery waste husks not only improved the nutrient profile and physicochemical properties of MSS-derived biochar but also mitigated heavy metal contamination. Additionally, this method reduced the presence of heavy metals, making it a more suitable alternative to biochar produced through monopyrolysis for use in agricultural applications.

## Graphical abstract

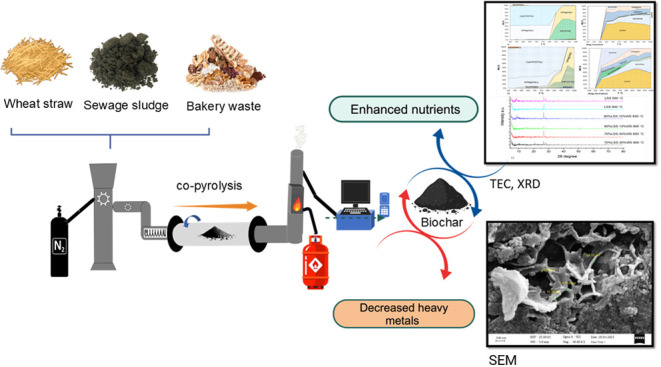

## Introduction

Phosphorus (P) is a crucial element for plant growth, contributing significantly to energy transformation^1,2^ and cellular reproduction.^3^ The ongoing depletion of P resources through extensive mining underscores the importance of developing efficient recovery methods.^1,4–6^ Municipal sewage sludge (MSS), a byproduct of wastewater processing, is a significant source of P,^7,8^ containing approximately 90% of the P found in wastewater.^2,9,10^ In addition to P, MSS is rich in organic matter and other nutrients, such as carbon (C) and nitrogen (N), rendering it a valuable resource for applications such as soil enhancement and livestock feed.^11,12^ However, in many countries, the utilization of MSS is prohibited because of the presence of organic^13^ and inorganic pollutants. These include various hazardous substances, including pathogens, polyfluoroalkyl substances (PFAS), antibiotics, UV filters, antiseptics, microplastics, phthalates, hormones, and heavy metals, which can contaminate soil and potentially enter the food chain.^14,15^

Thermal conversion methods for separating valuable components from harmful fractions include combustion, gasification, and pyrolysis.^16–18^ Pyrolysis processes offer several advantages. One significant benefit is its reduced emissions of COx, SOx, and NOx compared to combustion and gasification processes.^19,20^ Moreover, pyrolysis presents an alternative solution to landfilling, with lower greenhouse gas (GHGs) emissions.^21^ Pyrolysis converts organic waste into valuable products under high-temperature, oxygen-limited, or oxygen-free conditions.^22^ During pyrolysis, the organic components in sewage sludge decompose, yielding biochar, syngas, and oil/tar.^23^ Pyrolysis also decomposes most organic pollutants, including hormones and pathogens, and immobilizes or removes heavy metals.^24,25^ Furthermore, this process facilitates the recovery of essential nutrients in the biochar, enabling the resulting MSS biochars to be used as a soil conditioner^26^ or as a precursor for fertilizer production.^27–30^ While there are benefits to monopyrolysis of MSS, several operational challenges arise. MSS often contains heavy metals, which can persist in the biochar and pose environmental risks.^31^ In addition, studies have shown that P in MSS biochars typically exists in the form of aluminum (Al) and iron (Fe) phosphates, which are not readily available to plants.^32,33^ The low availability of P in these forms affects the plant growth. For instance, biochars with high Fe/Al–P content do not significantly enhance plant growth or P uptake compared to other forms of P fertilizers.^34^ This is because the P bound to Fe and Al is not easily accessible to plants, leading to reduced effectiveness as a soil conditioner.^33^ Other researches emphasized the importance of selecting the appropriate pyrolysis temperature to enhance P bioavailability, for example, pyrolysis temperatures above 600 °C result in the formation of more thermodynamically stable P species, such as apatite, which are less soluble and thus less immediately available in soil.^35–38^ This is because higher temperatures promote the conversion of organic P to more stable inorganic forms, such as calcium (Ca) phosphates. Similarly, Vali et al.^8^ showed that the feedstock type influences the formation of different P-bearing compounds during pyrolysis. For example, TEC revealed that P became more thermodynamically stable in species such as Si/Al­(PO_4_), CaP_2_O_7_, Ca_3_P_2_O_8_, and Mg_3_P_2_O_8_ at different temperatures above 500 °C, depending on whether the MSS was digested or undigested. Additionally, the study demonstrated that the Fe and Al phosphates present in sludge can be transformed into other P compounds, including inorganic P compounds and apatite.^35,39^ Co-treating sewage sludge with biomass or chemicals enhances the bioavailable P levels.^40^ Most additive treatments form complexes with Fe and Al, releasing P from Fe–P, Al–P, thereby enhancing P bioavailability.^41^ Research demonstrated that incorporating potassium (K) into sludge prior to pyrolysis substantially improved the accessibility of P in biochars.^42^ Chowdhury et al.^43^ demonstrated that the synergistic coapplication of biochar and K significantly enhances growth, physiological attributes, and antioxidant defense mechanisms in wheat under water-deficit conditions by restoring osmotic balance and improving photosynthetic efficiency. Consequently, it is necessary to develop and investigate new techniques that can stabilize heavy metals in biochar derived from MSS while improving the availability of P for plants over the medium term.

One innovative approach to producing tailored biochar for application in the soil conditioner is copyrolysis. Copyrolysis is similar to pyrolysis, except that it includes the simultaneous thermal decomposition of two or more feedstocks.^44^ Several investigations have found that copyrolysis can result in synergistic effects among the various components.^45^ For example, the copyrolysis of biomass and coal can accelerate the thermal breakdown of volatile chemicals and increase pyrolysis reactivity.^44^ Studies have shown that process parameters, such as pyrolysis temperature and feedstock mixing ratio, significantly impact the yield distribution and properties of pyrolysis products, making them suitable for specific applications in agriculture and environmental remediation.^45–47^ However, most studies have been using different biosolids and biomasses, and there is a lack of data regarding a blend ratio of feedstocks, such as different kinds of MSS and agricultural residue. The investigations of various feedstock combinations and their interactions under different pyrolysis conditions could provide valuable insights into the underlying mechanisms of synergy.^48^ The type of reactor is crucial in influencing copyrolysis behavior, as it affects particle contact and heat transfer.^46^ However, the majority of biosolids copyrolysis studies have been conducted using thermogravimetric reactors or fixed-bed-type reactors.^49^ These methods have limitations, including small sample sizes and slow heating rates, which may not accurately represent industrial-scale pyrolysis processes. To address these limitations, researchers have begun investigating the use of other types of reactors for biomass copyrolysis. Nonetheless, the copyrolysis of MSS with biomass in a rotary reactor has not been studied. Therefore, it is essential to investigate copyrolysis behavior in a rotary reactor and examine the biochar properties, as well as the fate of P and heavy metals, during the copyrolysis process.

This study aims to investigate the biochar properties and the fate of trace elements and P during the copyrolysis of MSS and agricultural residues at various temperatures. The rotary reactor was selected for this study due to its ability to provide uniform heating and effective mixing, which is crucial for ensuring consistent pyrolysis conditions when processing heterogeneous feedstocks, such as municipal sewage sludge and agricultural residues. These advantages make the rotary reactor a suitable choice for investigating biochar production while also serving as a foundation for scaling up pilot plant and industrial applications.^50^ The research focuses on examining how different temperatures, feedstock compositions, and mixing ratios affect the yield and quality of biochar as well as the behavior of heavy metals during the process. By combining experimental data with TEC, using calculation tools such as the combination of HSC chemistry and FactSage, this study seeks to provide a comprehensive understanding of the copyrolysis process and its potential to improve bioavailability in biochar. However, when wheat straw is added to two different types of MSS, significant changes occur in the levels of C, silicon (Si), K, chlorine (Cl), and ash content. These changes will be analyzed and interpreted in an experimental setup and by TEC, providing insights into future studies of pot experiments and bioavailability. The findings from this research will contribute to tailoring biochar to maximize the beneficial use of MSS and agricultural residues.

## Materials and Methods

### Copyrolysis Feedstocks

Two sludge samples were obtained from different wastewater treatment plants in Sweden, each utilizing distinct treatment and sludge processing methods. The samples were designated as BSS, representing anaerobically digested municipal sewage sludge from Bors WWTP (located at 57°43′16″N, 12°56′25″E), and LSS, representing undigested MSS from Lidköping WWTP (located at 58°29′53″N, 13°9′0″E), where P chemically precipitated using polyaluminum chloride. To minimize alterations in sludge properties and remove moisture prior to characterization, the samples were dried at 105 °C for 24 h in accordance with the ISO 11461 standards. Subsequently, the dried MSS samples were ground using a laboratory crushing mill and stored in sealed polyethylene containers before pyrolysis, resulting in MSS particles approximately 5 mm in size. Wheat straw (WS) and bakery waste (BKW) were procured from farms and bakeries in Sweden. The samples were air-dried and subsequently ground to a 5 mm thickness. To achieve thorough mixing, the materials were blended using a milling process for 15 min. Dissolution has been carried out with HNO_3_/HCl/HF according to SS EN 13656:2003 and ASTM D3682:2013 och ASTM D4503:2008. The total elemental composition of the samples was analyzed utilizing inductively coupled plasma-sector field mass spectrometry (ICP-SEMFS) in accordance with SS EN ISO 17294-2:2016 and EPA Method 200.8:1994. For chlorine analysis, the sample was sintered at 550 °C with Na_2_CO_3_ and ZnO, followed by water leaching and purification via cation exchange prior to analysis, adhering to SS EN ISO 17294-1,2 (mod) and EPA method 200.8 (mod).

### Copyrolysis Experiment

In this study, the copyrolysis samples comprised digested municipal sewage sludge (BSS) and undigested municipal sewage sludge (LSS) mixed with WS or BKW. The mixtures contained 90 or 70% municipal sewage sludge (either BSS or LSS) combined with 10 or 30% WS or BKW, respectively, on a dry weight basis. Dried samples were subjected to pyrolysis at three different temperatures (500, 650, and 900 °C), with each temperature tested in triplicate. The experiments employed a horizontal tube rotary pyrolysis reactor, Figure 1, which comprised a gastight feeding section, a pyrolysis section, and an end section equipped with a gas burner and a flue gas analyzer. The reactor was gradually heated to the target temperature, and nitrogen gas was introduced to create an inert atmosphere, ensuring optimal conditions for copyrolysis. The feeding process was initiated when the reactor reached the set temperature. MSS was fed into the rotary pyrolyzer using a screw grinder, conveyed through a feeding pipe, and placed in an electrically heated rotary oven with a rotating speed of 5 rpm. Water cooling systems were used to maintain low temperatures in the connection area. Uniform and rapid heating was achieved with an inner steel tube extending through the reactor, with temperatures monitored by using thermocouples. The semicontinuous process managed to convert 800 g of MSS into biochar per experiment. The resulting pyrolysis gases were combusted by using a supporting gas flame, and a Testo360 gas analyzer ensured complete combustion before ventilation. The system control adjusted the rates of the inlet gases accordingly. Air was supplied at a rate of 50,000 mL/min to cool the rotary feeder, while an additional 6,000 mL/min of air was used to combust the outlet gases. Nitrogen was introduced at a rate of 15,000 mL/min to transport the gases. Each experiment was replicated three times to ensure consistent results.

**Figure 1:**
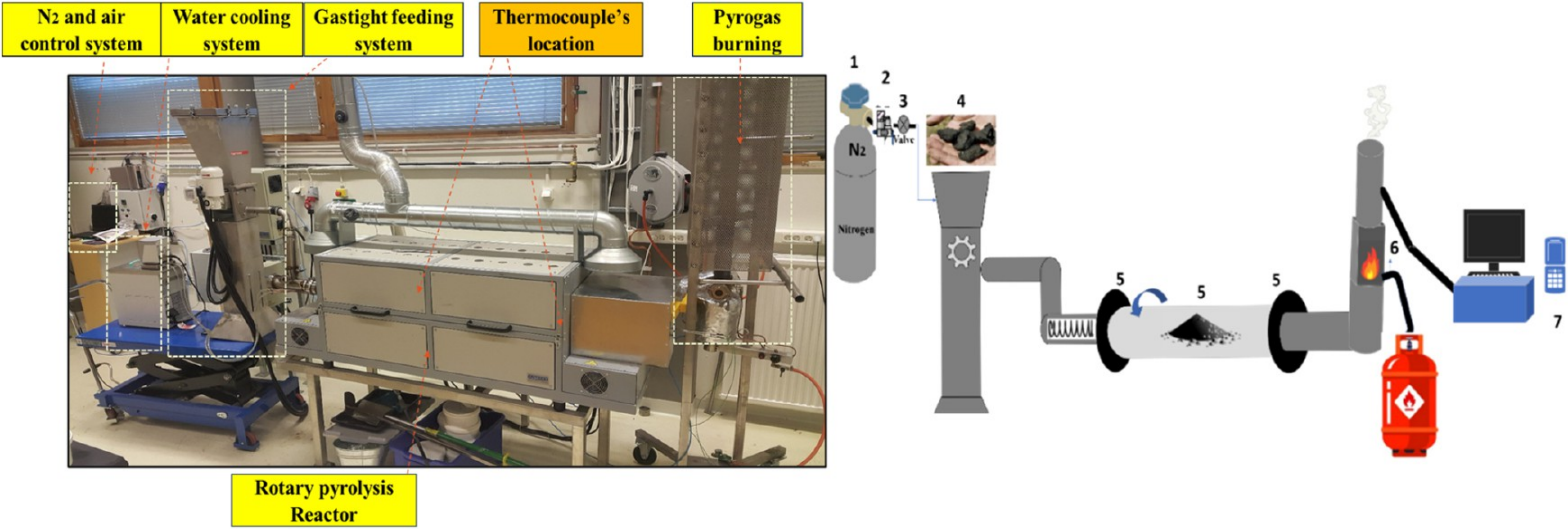
Lab scale rotating pyrolysis unit and its schematic drawing: (1) nitrogen, (2) valve, (3) pressure gage, (4) feeding system, (5) thermocouple, (6) gas burner, and (7) gas analyzer.

### Biochar Yield and Physicochemical Properties

#### Biochar Yield

The yield of biochar (*Y*_i_) was calculated as the ratio of the mass of the produced biochar to the mass of the initial pyrolysis feedstock, using eq 1:

1 $$\mathrm{biochar}\;\mathrm{yield}\;\left(\%\right)=\frac{\mathrm{mass}\;\mathrm{of}\;\mathrm{biochar}}{\mathrm{mass}\;\mathrm{of}\;\mathrm{feedstock}}\times 100$$

#### Total Concentration of Heavy Metals and Major Metals

To determine the total metal concentration in the biochar samples, 1 g of each sample was digested using an acid solution containing 6 mL of 65% nitric acid (HNO_3_), 2 mL of 37% hydrochloric acid (HCl), 1 mL of 30% hydrogen peroxide (H_2_O_2_), and 1 mL of 48% hydrofluoric acid (HF). The digestion process was conducted using a microwave digestion system (Milestone ETHOS UP microwave oven, Acquisition Corp., Hatfield, PA, USA) according to ISO 16967 and ISO 16968. Following digestion, the resulting solutions were filtered through a 0.20 μm filter and diluted to 50 mL with Milli-Q water. The concentrations of various elements in the biochar, including major elements (P, Mg, K, and Fe) and trace elements (Cd, Cu, Cr, Zn, and Pb), were analyzed using microwave plasma-atomic emission spectroscopy (MP-AES, Agilent Technologies, Santa Clara, CA).

#### Micromorphology and Surface Characterizations

The micromorphology and structural characteristics of biochar are crucial for understanding its potential applications, particularly in heavy metal stabilization. A detailed analysis of these properties provides insights into the efficiency and effectiveness of biochar as a soil amendment and pollutant adsorbent. The micromorphology of the biochar was examined by using an FESEM Sigma VP scanning electron microscope (ZEISS, Germany). The specific surface area, pore size, and pore volume were determined by N_2_ adsorption–desorption isotherm analysis using a BET surface area analyzer (BElSORP Mini, Microtrac Bel Corp).

#### Thermodynamic Equilibrium Calculations Modeling (TEC)

Thermodynamic calculations were conducted using FactSage 8.2,^51,52^ a comprehensive computational tool that includes various modules for accessing different solution databases and substances. The calculations are based on the Gibbs energy minimization principle to determine the quantities of each product at equilibrium.^52^

For the copyrolysis process, global multicomponent equilibrium calculations were conducted to investigate the fate of trace elements during copyrolysis of different feedstocks. One limitation of thermodynamic calculation modeling is the limited availability of compound data, especially when dealing with complex systems, such as the thermal conversion of biomass and sewage sludge, which contain numerous elements. To address this limitation, this study utilized the thermodynamic databases FactPS, FToxid, and FTsalt, along with the HSCA database from HSC Chemistry 10.^53,54^ The HSCA database contains compounds including trace elements, such as As, Co, Cr, Cu, Mn, Mo, Ni, Pb, V, and Zn, thereby enhancing the accuracy of the calculations for these elements.^55^ Additionally, the database includes compounds of major elements, such as Al, Ca, Fe, K, Mg, Na, and Si. However, for P species and to evaluate the effect of adding wheat straw, thermodynamic equilibria were calculated using FactSage software version 7.3.^52^ The GTOX (stoichiometric compounds, solutions, and gas species) and the SGPS (SGTE pure substance database) were used to implement the thermodynamic data for gaseous compounds. It is worth mentioning that based on literature review, GTOX contains more P-containing compounds associated with major ash-forming elements, such as K and Ca, compared to FToxid.^56^ In the case of gases and stoichiometric compounds which are duplicated in both SGPS and the GTOX, the species of SGPS was suppressed and they were selected from the GTOX. Nitrogen (N_2_) was assumed to act as an inert gas during the pyrolysis process calculations, which were conducted at a pressure of 1 atm and a temperature range of 500–1000 °C.

## Results and Discussion

### Analytical Properties of the Raw Copyrolysis Feedstock

The chemical characteristics of the raw feedstocks used in this study are presented in Table 1. The primary differences between the MSSs are carbon, ash, moisture, and P contents, as they have undergone different processes in WWTPs and the different feedstocks. For instance, LSS had a significantly higher carbon content (445 g/kg TS) compared to BSS (294 g/kg TS), as some C was released as methane gas during the aerobic digestion of BSS. This indicates that LSS retains more organic matter and potentially has a higher energy content, which could influence the biochar yield during pyrolysis. BSS had a significantly higher ash content (400 g/kg TS) than LSS (216 g/kg TS), suggesting that BSS underwent more decomposition and concentration of inorganic components, potentially leading to different ash characteristics during pyrolysis. Additionally, BSS exhibited a higher P content (29 g/kg TS) than LSS (18 g/kg TS), which renders BSS more promising for P recovery during copyrolysis. WS is notable for its particularly high K content (6.1 g/kg TS) compared to the relatively low levels found in sewage sludges (LSS: 2.1 g/kg TS, BSS: 2.5 g/kg TS) and bakery waste (BKW: 3.5 g/kg TS). The high K content in WS could enhance the nutrient value of biochar as a soil amendment, particularly for crops that require K. Conversely, the Si content was especially high in wheat straw (16 g/kg TS) compared to the lower levels found in bakery waste. According to the literature,^57^ this high Si concentration is advantageous because it can enhance the thermal stability of biochar and act as a catalyst during pyrolysis, promoting the retention of P and heavy metals. However, the chlorine content of WS (3 g/kg of TS) was considerably higher than in the other feedstocks, which could present operational challenges. One of the challenges during monopyrolysis of WS and BKW is the high production of bio-oil and gas, which are associated with the high volatile matter content of these raw feed materials. In addition, monopyrolysis of MSS often encounters issues with dust formation, while WS pyrolysis faces challenges, such as tar formation and feeding difficulties. These operational challenges were mitigated by copyrolyzing MSS with WS and BKW.^58^ Pyrolysis of agricultural residues produces tar, a complex mixture of organic compounds, including oxygenated and aromatic hydrocarbons, as well as sulfur-containing compounds.^59^ Tar-cracking reactions break down heavier hydrocarbons into lighter gases, such as carbon monoxide (CO), methane (CH_4_), and hydrogen (H_2_). The addition of biomass with lower ash content and different physical properties helps alleviate dust-related issues in MSS, leading to improved feeding and handling during pyrolysis.^60^

**Table 1:** Elemental Content and Characteristics of the Raw Samples

parameter	LSS	BSS	bakery waste	wheat straw	unit	method
moisture (as received)	71 ± 0.1	73 ± 0.2	8.8 ± 0.1	7.52 ± 0.5	wt %	I-WT-CC
carbon	445 ± 0.9	294 ± 0.9	459 ± 0.8	454 ± 0.3	g/kg TS^a^	I-ELEM-TCDS
hydrogen	65.1 ± 0.2	53.0 ± 0.5	60.4 ± 0.7	4.8 ± 0.4	g/kg TS	I-ELEM-TCDS
oxygen	226 ± 0.5	218 ± 0.4	475 ± 0.5	531 ± 0.5	g/kg TS	I-ELEM-TCDS
nitrogen	42.2 ± 0.1	30.0 ± 0.1	6.40 ± 0.1	6.32 ± 0.1	g/kg TS	I-ELEM-TCDS
sulfur	6.75 ± 0.1	11.0 ± 0.1	0.805 ± 0.1	2.45 ± 0.1	g/kg TS	I-ELEM-TCDS
chlorine	0.301 ± 0.01	0.201 ± 0.09	2.12 ± 0.1	3.00 ± 0.1	g/kg TS	ICP-SFMS
ash content in dry matter	216 ± 0.5	400 ± 0.03	46.4 ± 0.3	56.0 ± 0.1	g/kg TS	I-ASH550GRS
Ash Analysis						
Al	38.1 ± 6.7	81.0 ± 6.7	0.205 ± 0.5	0.364 ± 0.4	g/kg TS	ICP-SFMS
Ca	9.30 ± 1.2	17.0 ± 2.1	0.907 ± 0.1	2.91 ± 0.1	g/kg TS	ICP-SFMS
Fe	6.90 ± 1.0	16.0 ± 1.0	0.307 ± 0.5	0.117 ± 0.2	g/kg TS	ICP-SFMS
K	2.10 ± 0.5	2.54 ± 0.5	3.50 ± 0.5	6.16 ± 1.5	g/kg TS	ICP-SFMS
Mg	1.60 ± 0.6	3.15 ± 0.6	0.907 ± 0.2	0.560 ± 0.3	g/kg TS	ICP-SFMS
Na	1.65 ± 0.2	1.55 ± 0.2	2.10 ± 0.4	0.504 ± 1.5	g/kg TS	ICP-SFMS
P	17.5 ± 2.7	29.2 ± 2.7	1.60 ± 0.02	0.784 ± 0.2	g/kg TS	ICP-SFMS
Si	18.2 ± 3.2	33.3 ± 3.2	13.2 ± 0.2	16.8 ± 1.5	g/kg TS	ICP-SFMS
Trace Elements						
As	3.20 ± 1.3	3.10 ± 0.7	3.00 ± 0.9	0.056 ± 0.1	mg/kg TS	ICP-SFMS
Cd	0.301 ± 0.1	0.601 ± 0.2	0.0405 ± 0.1	0.0253 ± 0.2	mg/kg TS	ICP-SFMS
Co	2.22 ± 0.5	3.71 ± 1.6	0.190 ± 0.1	0.0121 ± 0.1	mg/kg TS	ICP-SFMS
Cr	25.4 ± 5	33.2 ± 11	0.414 ± 0.1	0.137 ± 0.1	mg/kg TS	ICP-SFMS
Cu	140 ± 5	280 ± 5	6.22 ± 0.1	1.21 ± 0.2	mg/kg TS	ICP-SFMS
Ni	9.22 ± 2.1	15.4 ± 3.5	1.02 ± 0.7	0.0547 ± 0.1	mg/kg TS	ICP-SFMS
Mn	159 ± 28	397 ± 73	41.5 ± 8.1	13.1 ± 0.3	mg/kg TS	ICP-SFMS
Pb	7.42 ± 1.8	17.1 ± 4.0	0.912 ± 0.2	0.0705 ± 0.01	mg/kg TS	ICP-SFMS
Zn	200 ± 5	600 ± 10	18.1 ± 0.4	9.33 ± 0.01	mg/kg TS	ICP-SFMS

a TS: total solid.

### Biochar Characteristic

#### Basic Properties

The yield percentages of different biochars are listed in Table 2. The data indicate that biochar yield decreases with temperature increases across all feedstock combinations. For example, the 90 wt % LSS + 10 wt % BKW showed a yield of 36% at 500 °C, which dropped to 30% at 900 °C. This trend suggests that higher temperatures lead to more complete pyrolysis, thereby reducing the yield of biochar. Research has reported the compositions of cellulose, hemicellulose, and lignin in WS to be 42–50, 23–30, and 16–18 wt %, respectively.^45,61^ This indicates that the high volatile matter and low ash content in WS and BKW contribute to greater oil and gas yields during the pyrolysis of lignocellulosic biomass compared to MSS, which is primarily composed of proteins, fats, cellulose, and carbohydrates.^62^ When BKW and WS are used in copyrolysis, the biochar yield decreases. Furthermore, as the proportion of WS and BKW increases, the yield declines at every temperature. This reduction is the result of introducing a lower amount of ash content and metals alongside the increased agricultural residues compared to MSS. Studies have also found that copyrolysis helps balance the yield of oil and gas, reducing excessive volatile matter release while maintaining favorable biochar properties.^45,47^ Additionally, the P concentration increases with temperature in all cases. BSS-based mixtures demonstrate the highest P content, with the 90% BSS-10% BKW mixture yielding up to 55 g/kg at 900 °C. Different LSS-based biochars display lower P levels, with the 90% LSS-10% BKW biochar reaching a maximum of 39 g/kg at 900 °C. Fe content is generally higher in BSS biochars, with the 90% BSS-10% WS exhibiting a significant increase in Fe levels at 900 °C (21 g/kg). Conversely, the magnesium content is the highest in WS biochars, particularly in the 70% LSS-30% WS biochar, which contains up to 1.8 g/kg Mg at 900 °C. K content is notably higher in WS compared to BKW biochars. For instance, 70% LSS-30% WS biochar contains 12 g/kg of K at 900 °C, while 90% LSS-10% BKW biochar contains only 5.4 g/kg at the same temperature. The retention of K in biochars aligns with findings from other studies, which indicate that adding MSS to WS during cocombustion enhances K retention in the ash by forming high-melting-point compounds, such as K aluminosilicates and alkali phosphates.^11^ The results displayed in Table 2 align with previous research, indicating that several nutrients and metals, such as P, K, Mg, and Fe, concentrate in biochar during the copyrolysis process.^11,63,64^

**Table 2:** Yield Percentage of Different Biochars and the Main Nutrients

	*T*, °C	biochar yield, %	P	Fe	Mg	K	unit
90%LSS-10%BKW	500	36 ± 3	33.5 ± 2.4	13.2 ± 0.5	0.412 ± 0.02	4.91 ± 0.3	g/kg biochar
	650	33 ± 4	37.5 ± 1.5	13.2 ± 0.4	0.346 ± 0.01	5.10 ± 0.5	g/kg biochar
	900	30 ± 3	39.1 ± 1.5	10.9 ± 0.3	0.214 ± 0.01	5.40 ± 0.4	g/kg biochar
70%LSS-30%BKW	500	30 ± 3	23.6 ± 2.4	7.64 ± 0.4	0.415 ± 0.05	5.45 ± 0.05	g/kg biochar
	650	28 ± 2	24.2 ± 2.0	7.35 ± 0.5	0.447 ± 0.04	4.38 ± 0.04	g/kg biochar
	900	25 ± 1	27.5 ± 2.0	8.01 ± 0.5	0.764 ± 0.04	4.55 ± 0.02	g/kg biochar
90%LSS-10%WS	500	38 ± 3	28.6 ± 1.2	8.60 ± 0.4	0.825 ± 0.02	5.15 ± 0.03	g/kg biochar
	650	36 ± 1	29.5 ± 1.7	9.10 ± 0.2	0.814 ± 0.04	6.44 ± 0.02	g/kg biochar
	900	32 ± 3	30.3 ± 2.5	9.40 ± 0.2	0.885 ± 0.04	7.76 ± 0.05	g/kg biochar
70%LSS-30%WS	500	34 ± 5	22.6 ± 1.0	8.80 ± 0.3	1.43 ± 0.04	7.25 ± 0.07	g/kg biochar
	650	33 ± 2	23.4 ± 1.0	8.50 ± 0.5	1.45 ± 0.02	9.36 ± 0.07	g/kg biochar
	900	29 ± 1	27.1 ± 4.0	8.40 ± 0.5	1.87 ± 0.01	12.6 ± 0.09	g/kg biochar
90%BSS-10%BKW	500	50 ± 5	46.2 ± 2.5	13.1 ± 0.3	0.447 ± 0.05	5.21 ± 0.02	g/kg biochar
	650	48 ± 3	48.2 ± 3.0	15.3 ± 0.5	0.552 ± 0.08	5.30 ± 0.05	g/kg biochar
	900	44 ± 4	55.2 ± 1.5	16.5 ± 0.3	0.414 ± 0.05	6.40 ± 0.02	g/kg biochar
70%BSS–30%BKW	500	47 ± 4	39.5 ± 2.0	13.9 ± 0.4	2.32 ± 0.03	5.20 ± 0.04	g/kg biochar
	650	43 ± 3	40.1 ± 3.0	14.3 ± 0.5	4.75 ± 0.06	4.60 ± 0.05	g/kg biochar
	900	39 ± 2	45.1 ± 2.5	14.7 ± 0.3	7.28 ± 0.09	6.25 ± 0.06	g/kg biochar
90%BSS-10%WS	500	50 ± 5	44.3 ± 2.0	13.3 ± 0.5	0.932 ± 0.08	3.84 ± 0.02	g/kg biochar
	650	47 ± 4	46.8 ± 3.1	16.1 ± 0.3	0.852 ± 0.05	3.96 ± 0.05	g/kg biochar
	900	45 ± 3	49.5 ± 1.5	21.2 ± 0.5	0.814 ± 0.06	4.21 ± 0.03	g/kg biochar
70%BSS–30%WS	500	46 ± 5	39.1 ± 1.5	12.1 ± 0.4	0.625 ± 0.08	4.95 ± 0.06	g/kg biochar
	650	42 ± 2	40.5 ± 1.7	15.1 ± 0.7	0.547 ± 0.06	5.23 ± 0.05	g/kg biochar
	900	40 ± 1	42.8 ± 1.5	17.5 ± 0.5	0.635 ± 0.08	5.94 ± 0.06	g/kg biochar
LSS	500	41 ± 4	29.6 ± 0.06	13.1 ± 0.5	0.391 ± 0.02	9.11 ± 0.03	g/kg biochar
	650	34 ± 3	34.8 ± 0.08	14.1 ± 0.6	0.356 ± 0.05	5.64 ± 0.04	g/kg biochar
	900	31 ± 1	32.3 ± 0.08	17.1 ± 0.8	0.379 ± 0.04	6.52 ± 0.05	g/kg biochar
BSS	500	61 ± 1	39.1 ± 0.4	21.4 ± 0.2	0.356 ± 0.03	4.53 ± 0.03	g/kg biochar
	650	52 ± 2	40.7 ± 0.7	22.5 ± 0.4	0.317 ± 0.04	4.37 ± 0.05	g/kg biochar
	900	47 ± 2	53.1 ± 0.6	23.1 ± 0.3	0.476 ± 0.07	4.66 ± 0.02	g/kg biochar

#### Heavy Metals (HMs) Analysis of Biochars

Figure 2 presents the concentrations of various heavy metals in biochar produced from the pyrolysis of different mixtures of WS and BKW with MSSs. The metal concentrations in biochar vary depending on the temperature and feedstock mixtures. The mechanism of HMs retention in biochar during copyrolysis, mainly driven by the devolatilization of organic matter, aligns with findings from other studies.^47^ The concentration of Cd is nearly undetectable in all biochars. The volatilization of Cd at pyrolysis temperatures higher than 500 °C is consistent with findings that Cd is a volatile element, and its release is influenced by the pyrolysis temperature.^65,66^ Since Cd volatilizes at temperatures above 500 °C, it is likely to be present in the flue gas. Therefore, effective flue gas cleaning systems are essential to capture Cd and prevent its release into the environment.^65^

**Figure 2:**
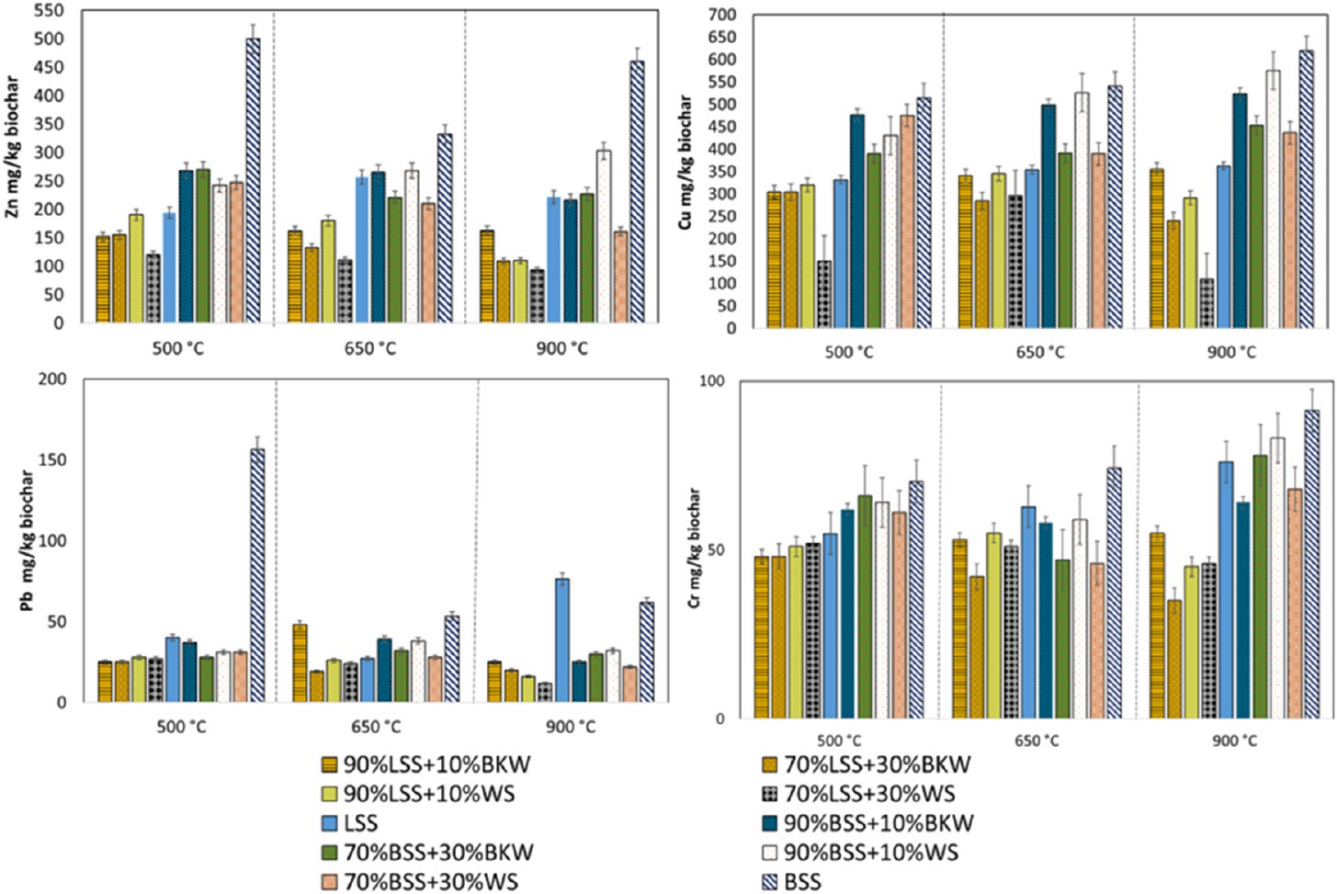
Heavy metal concentration in biochars.

As illustrated in Figure 2, the concentration of HMs in sewage sludge biochar increases following pyrolysis, with higher temperatures resulting in a higher HMs concentration in the biochar. This phenomenon is primarily attributed to the significant mass reduction of organic compounds, which leads to the enrichment of HMs within the biochar matrix. Given that HMs exhibit higher thermal stability compared to organic matter, which decomposes and volatilizes at elevated temperatures, they remain concentrated in the biochar.^67^ The retaining trend of HMs in biochar follows the order: Cr > Cu > Zn > Pb. Regarding HMs concentrations after copyrolysis with different feedstocks, biochars derived from BSS generally contain higher metal concentrations than those from LSS-based mixtures. This disparity can be attributed to the inherently higher ash content of BSS, resulting from anaerobic digestion in the WWTP. Furthermore, as the proportion of WS and BKW in the feedstock increased, the initial HMs concentrations decreased in the feedstocks, thereby explaining the lower HMs content observed in copyrolyzed biochar compared to monopyrolyzed biochar. Consistent with findings from another study,^47^ the copyrolysis of biosolids with lignocellulosic biomass has been shown to effectively reduce heavy metal concentrations in the resulting biochar. This suggests that the addition of WS enhances Zn and Pb removal at higher temperatures for both digested and undigested MSS, potentially decreasing the environmental risks resulting from the elevated concentrations of HMs in MSS biochars. These findings are consistent with previous studies, which reported that chlorine (present in WS) facilitates Zn removal.^68^ Furthermore, another study demonstrated that copyrolyzing sewage sludge with metal-loaded poly­(vinyl chloride) microplastic significantly improves heavy metal stabilization and reduces biochar bioavailable contents of Cd, Cr, Ni.^69^ Similarly, in this study, the higher chlorine content present in WS and BKW (compared to MSS) contributed to the removal of Zn and Pb. Among the HMs examined, Pb showed a noticeable reduction with the addition of WS and BKW during copyrolysis, especially at higher temperatures. However, the elevated chlorine levels also introduced challenges, including agglomeration, corrosion, and other operational issues, as is commonly observed when copyrolyzing with chloride-rich materials.^70^ Cu retention generally increased as the temperature rose from 500 to 900 °C. The mixture containing 90% BSS and 10% WS showed the highest Cu concentration at 650 °C, with stability maintained at 900 °C. Additionally, the initial concentration of Cu in the feedstock decreased as the amount of WS increased, which may explain the observed decrease in Cu levels at higher temperatures. Cr, on the other hand, exhibited a high degree of retention across all temperature ranges. Additionally, the increase in Cr content at a temperature of 900 °C could be attributed to contamination, possibly due to Cr leaching from the reactor’s pipes during the pyrolysis.

#### Morphological and Physicochemical Properties

Table 3 displays the physicochemical properties of various biochars at 650 and 900 °C, as shown from BET analysis, for both pyrolyzed MSSs and copyrolyzed biochars. The data demonstrate the significant impact of pyrolysis temperature and feedstock composition on the surface area, pore size, and pore volume of biochar. Increasing the pyrolysis temperature from 650 to 900 °C, as well as the addition of WS and BKW, enhances the surface area across almost all feedstock combinations. This can be attributed to the high release of volatile matter at higher temperatures, which is consistent with previous studies that link this increase to enhanced thermal decomposition and volatile release.^47^ Biochars derived from BSS exhibited higher surface areas than those derived from LSS, likely due to the pretreatment effect of anaerobic digestion in BSS. This process breaks down organic matter and creates a more porous, stable, fixed carbon structure, while LSS-derived biochars may transition into a graphite-like microcrystalline structure.^71^

**Table 3:** BET Surface Area of Biochar Produced from Pyrolysis/Copyrolysis at Different Temperatures

	*T*, °C	surface area (m^2^/g)	avg. pore size diameter (nm)	pore vol. (cm^3^/g)
90%LSS-10%BKW	650	37.86	14.76	0.139
	900	60.61	7.343	0.251
70%LSS-30%BKW	650	40.36	11.36	0.159
	900	68.84	9.710	0.162
90%LSS-10%WS	650	20.53	15.32	0.0780
	900	117.4	5.197	0.152
70%LSS-30%WS	650	25.40	14.08	0.0890
	900	150.8	4.323	0.163
90%BSS-10%BKW	650	27.77	17.54	0.122
	900	107.2	6.796	0.182
70%BSS–30%BKW	650	73.32	10.23	0.187
	900	108.2	6.134	0.165
90%BSS-10%WS	650	67.87	10.25	0.174
	900	125.2	7.688	0.245
70%BSS–30%WS	650	76.86	5.962	0.114
	900	74.36	7.691	0.143
LSS	700	38.06	14.85	0.145
	900	66.90	7.546	0.175
BSS	700	78.68	11.13	0.217
	900	127.3	7.626	0.249

Copyrolysis with WS significantly improved the surface area compared to BKW mixtures, particularly at higher temperatures (except for LSS-mixtures at 650 °C and for the BSS-mixtures at 900 °C). This improvement can be attributed to the higher volatile matter content in WS compared to BKW. However, some copyrolysis biochars, especially those with BKW, showed slightly lower surface areas than biochars produced from single MSS pyrolysis. This may be due to tar formation, which can block biochar pores, as observed by Wang et al.^46^ The reduction in average pore size at higher temperatures further supports the formation of a more refined, microporous structure, enhancing the material’s potential for application of heavy metal absorption and soil remediation.^72,73^ Trends in the pore volume varied with different feedstock mixtures but generally increased with rising temperatures. For example, the 90% LSS + 10% BKW mixture showed a notable increase in pore volume, from 0.13 cm^3^/g at 650 °C to 0.25 cm^3^/g at 900 °C, indicating greater porosity and enhanced gas permeability at elevated temperatures. This structural change, particularly in biochars derived from WS mixtures, plays a crucial role in improving the material’s adsorption capacity and the potential for heavy metal stability and soil remediation. While both WS and BKW contribute to these enhancements, WS has a more pronounced influence on surface area and pore structure development, making it a more effective feedstock for creating highly porous biochar for environmental applications.

The SEM images of biochar produced from various feedstocks are presented in Figures 3 and 4. When the single MSS-derived biochar at 650 and 900 °C is compared to the biochar samples where 10 and 30% BKW and WS were added, a clear distinction in surface texture and particle distribution can be observed. LSS-derived biochar appears to have a more aggregated and amorphous surface, indicating lower porosity and surface area, which aligns with the lower surface area values reported in Table 3 for LSS (38 m^2^/g at 700 °C). However, when BKW and WS were added, particularly at 900 °C, the biochar’s structure became more porous and granular, with noticeable fine particles uniformly distributed on the surface. This enhanced porosity is especially visible in samples such as 90% LSS + 10% WS and 90% BSS + 10% WS at 900 °C, where a more ordered and granular surface texture is evident. In contrast, at 650 °C, the biochar produced from BSS mixed with BKW (as seen with 10 and 30% additions) shows a granular, highly clustered morphology with a fine particle distribution. This indicates incomplete decomposition at lower temperatures, where the structure retains some complexity. The images highlight smaller, spherical agglomerates and visible porosity, which become more pronounced as the temperature increases to 900 °C. The pores appear more developed, suggesting enhanced gas release at higher temperatures, while the BKW mixtures help form a more structured matrix. This is consistent with the increase in BET surface area, where biochar from a 70% digested sewage sludge +30% BKW mixture shows an increase from 73.3 m^2^/g at 650 °C to 108.1 m^2^/g at 900 °C, reflecting improved porosity and surface area development at higher temperatures.

**Figure 3:**
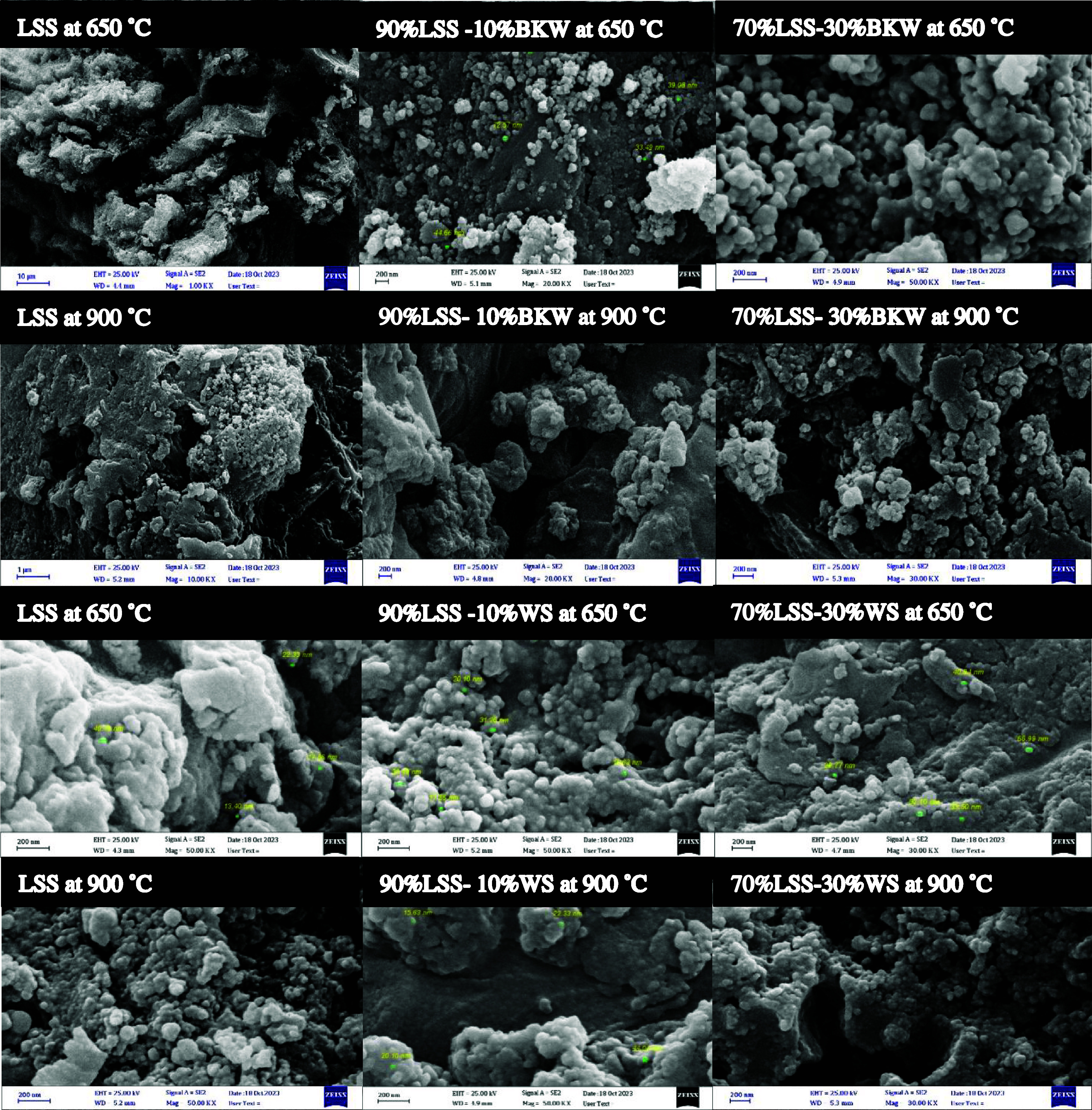
SEM images at various temperatures and mixing ratios for undigested sewage sludge, LSS. The scale bar indicates a margin size of 200 nm.

**Figure 4:**
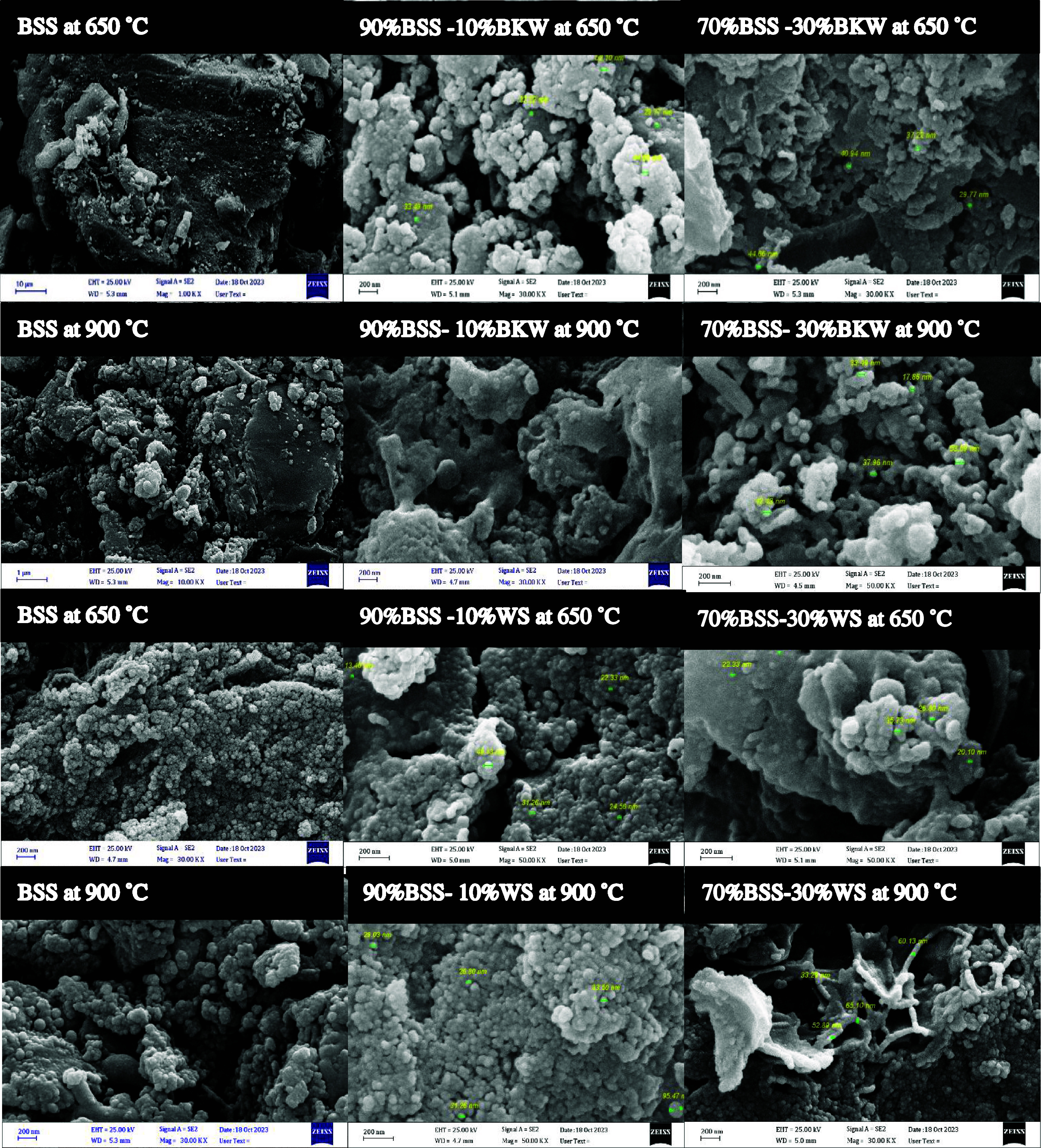
SEM images at various temperatures and mixing ratios for digested sewage sludge, BSS. The scale bar indicates a margin size of 200 nm.

The visible enhancement in the pore structure and particle distribution at higher temperatures, as seen in the SEM images, directly correlates with these increased surface area values. These structural changes enhance the biochar’s ability to trap and stabilize HMs such as Zn, Cr, and Cu. Regarding heavy metal stabilization, the SEM images also suggest that the addition of BKW and WS to the biochar matrix improves the metal retention. The finer particle distribution observed in the images may provide more active sites for the HMs stability. This is supported by the data on metal retention in Figure 2, which shows improved Zn and Cu retention in biochar produced at 900 °C with WS, a feedstock high in Si and Cl content. The uniform distribution of smaller particles, especially in the WS-amended samples, likely facilitates a better interaction between the biochar matrix and the HMs, leading to enhanced stabilization in the biochar matrix.

#### H/C and C/N Ratio of Produced Biochars

Table 4 represents the C, H, and N contents in biochar on a dry basis. The decrease in C, H, and N content in biochar compared to their feedstock blends during both pyrolysis and copyrolysis aligns with previous studies.^45,46^ It is deemed to be attributed to the thermal decomposition of organic matter, which breaks down volatile components and increases the ash content in the biochar. As shown in Table 4, biochars produced at lower temperatures (e.g., 500 °C) tend to retain more C and H than those produced at higher temperatures, such as 900 °C. For instance, in the 70 wt % LSS-30% BKW-derived biochars, the C content decreases from 47.2 wt % at 500 °C to 45.7 wt % at 900 °C. Similarly, hydrogen content also decreases with temperature, dropping from 2.5 to 1.9 wt % in the same mixture, reflecting a general trend across different feedstock combinations, where carbonization intensifies at higher temperatures. Furthermore, blending lignocellulosic biomass, such as WS or BKW, with MSS can increase the resulting biochar’s carbon and hydrogen content. For example, the 70% LSS-30% WS derived biochars contain 52.1 wt % carbon at 500 °C, which is notably higher than the carbon content of the 90% LSS-10% WS derived biochars at the same temperature (44.2 wt %). This enhancement makes biochar from WS or BKW copyrolysis a promising option for agricultural soil amendment. Conversely, biochar produced from anaerobically digested sludge tends to have lower C and H levels. For instance, 90% BSS-10% BKW-derived biochars at 500 °C show a carbon content of just 30.1 wt %, in contrast to the higher-carbon biochars from undigested sludge. This difference is likely due to the lower organic content in anaerobically digested sludge, which results in higher ash fractions and lower overall carbon and hydrogen levels compared to those in the untreated sludge. The H/C ratio, which signifies the level of aromaticity in biochar, also decreases as the pyrolysis temperature rises, indicating greater structural stability and aromaticity at higher temperatures. For instance, the 70% LSS-30% WS derived biochars show a decrease in the H/C ratio from 0.05 at 500 °C to 0.01 at 900 °C. A low H/C ratio is desirable in biochar, as it reflects a more stable structure, making it suitable for long-term carbon sequestration in soils, since lower H/C ratios in biochar also enhance its resistance to microbial degradation.^74,75^ However, biochars from copyrolysis with BSS tend to have higher H/C ratios, suggesting relatively lower carbon stability in those biochars. Across different feedstock combinations, it is observed that as the pyrolysis temperature increases, nitrogen is increasingly released, enhancing the stability of the biochar but reducing its nutrient content. Lower nitrogen levels in biochar can be advantageous for long-term nutrient storage, but it may limit its immediate nutrient value when applied as a soil amendment.^76–78^

**Table 4:** C/H/N Contents in Biochars at Various Temperatures from Pyrolysis and Copyrolysis Experiments on a Dry Basis

	*T*, °C	C (mol %)	H (mol %)	N (mol %)	H/C
90%LSS-10%BKW	500	3.3	2.6	0.29	0.80
	650	3.2	1.7	0.24	0.54
	900	2.9	1.6	0.25	0.55
70%LSS-30%BKW	500	3.9	2.5	0.29	0.64
	650	4.0	1.6	0.21	0.40
	900	3.8	1.9	0.26	0.50
90%LSS-10%WS	500	3.7	2.4	0.29	0.65
	650	3.4	1.6	0.24	0.47
	900	3.0	0.6	0.06	0.20
70%LSS-30%WS	500	4.3	2.6	0.27	0.60
	650	4.0	1.6	0.22	0.40
	900	3.8	0.7	0.09	0.19
90%BSS-10%BKW	500	2.5	1.9	0.16	0.76
	650	2.1	1.3	0.11	0.62
	900	2.3	1.1	0.13	0.47
70%BSS–30%BKW	500	2.9	2.3	0.17	0.78
	650	2.7	1.4	0.10	0.52
	900	2.1	0.5	0.04	0.24
90%BSS-10%WS	500	2.4	2.1	0.18	0.86
	650	2.3	1.1	0.11	0.47
	900	1.8	0.49	0.04	0.27
70%BSS–30%WS	500	2.9	1.8	0.15	0.61
	650	2.8	1.5	0.10	0.53
	900	2.8	0.50	0.05	0.18
LSS	500	3.5	3.7	0.37	1.06
	650	3.1	1.8	0.24	0.58
	900	2.5	4.9	0.09	1.96
BSS	500	3.4	7.4	0.12	2.21
	650	2.9	6.6	0.11	2.26
	900	2.3	5.6	0.09	2.40

### Phosphorus Speciation by Modeling and X-ray Diffraction (XRD)

The results of the thermodynamic equilibrium calculations, focusing on P compounds during the copyrolysis of MSS and WS, slag development, and XRD analysis on biochars at different temperatures are presented in Figure 5. The GTOX and SGPS databases were employed to predict the fate of P, focusing on the effect of WS when mixed with LSS as a representative.

**Figure 5:**
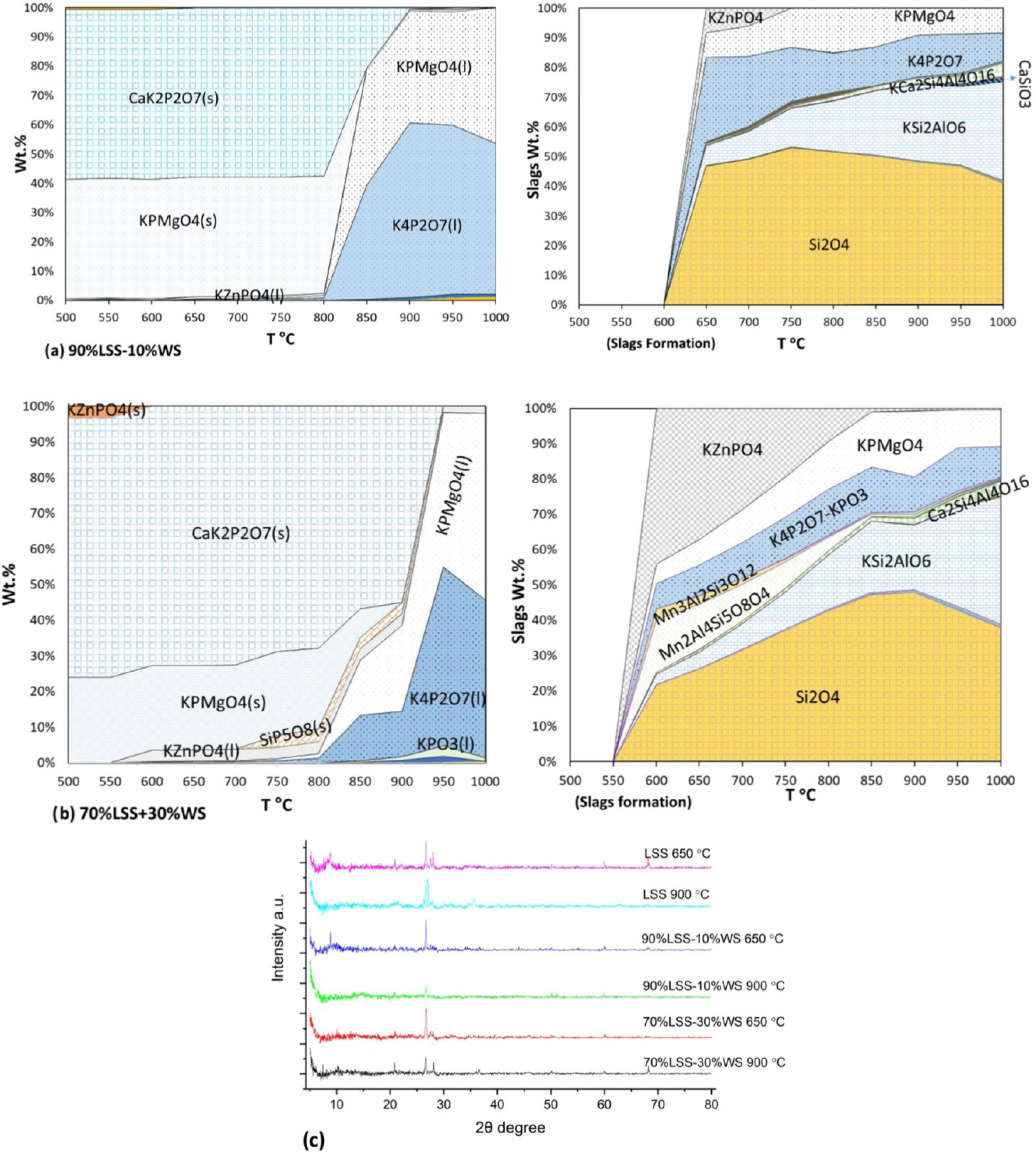
Calculated P-bearing compounds (left) and calculated slag formation (right) during copyrolysis at various temperatures: (a) 90% LSS-10% WS, (b) 70% LSS-30% WS, and (c) XRD.

In our last study 8, the TEC for monopyrolysis of LSS predicted that pure LSS biochar may contain high levels of Fe_2_O_3_, Al_2_O_3_, and Si/AlPO_4_, with slag formation potentially starting at 700 °C. The TEC results for copyrolysis of LSS with 10% WS (Figure 5a) indicate that Si_2_O_4_ becomes the dominant phase above 600 °C, forming stable structures and accounting for nearly 50 wt % of the predicted slag. Increasing the WS content to 30 wt % (see Figure 5b) further promotes the formation of calcium silicate at lower temperatures attributed to the increased K content in the system. The predicted formation of K-containing phosphates suggests their role in stabilizing P and certain heavy metals, particularly Zn. Additionally, at 600 °C, TEC predicts the emergence of Mn_3_(Al_4_Si_5_O_22_) and MnZn­(Al_4_Si_5_O_18_), which could aid in stabilizing Mn and Zn within the biochar. After the temperature reaches 700 °C, K-based compounds such as K_4_P_2_O_7_ and KZnPO_4_ are predicted to form, which could contribute to the retention of P and Zn. At 600 °C, KPMgO_4_ is thermodynamically stable, suggesting its potential presence at all temperatures. The TEC predictions indicate a strong affinity of P for K, leading to the formation of K phosphates. Furthermore, K and Si from WS are expected to play significant roles in the retention of P by forming stable phosphate phases, promoting slag formation, and potentially aiding in the capture of heavy metals such as Zn and Mn. Additionally, KZnPO_4_ and KPMgO_4_ are predicted to form at higher temperatures, which could stabilize metals such as Zn and Mg in phosphate phases. This predicted stabilization may have implications for soil remediation, as literature suggests that P–K–Mg compounds are more bioavailable to plants.^79^

The XRD analysis (Figure 5c) was conducted to identify various crystalline materials that impact the properties of biochar for its potential applications as a soil conditioner. Carbon in biochar typically exhibits broad peaks between 20 and 30° (2θ) in XRD, with the peak position and width reflecting the degree of graphitization.^80^ As the pyrolysis temperature increases, the graphitic carbon crystallite size also increases.^81^ Comparing the pattern with other studies^82^ reveals that KMgPO_4_ potentially can be recognized in the 25–30° (2θ) and 30–35° (2θ) peak ranges. However, at higher temperatures, as a result of slag formation, this compound becomes amorphous with decreased crystallinity and less defined peaks (see Figure 5c). In pure LSS biochar, especially at 650 °C, AlPO_4_ can be observed at peaks around 22, 27, and 42°.^80^

### Effect of Copyrolysis on the Fate of Heavy Metals

Figure 6 illustrates the partitioning behavior of Pb in gas, liquid, and biochar, as well as its predicted products, during the copyrolysis of LSS-WS mixtures at temperatures ranging from 500 to 1000 °C. The data showing the calculated compounds are based on TEC with the feedstock compositions as input. The calculations were performed using FactSage software for calculations incorporating a combination of databases, FactPS, FToxid, and FTsalt, along with the HSCA database from HSC Chemistry 10. These databases were employed to predict the fate of HMs, with a particular focus on the effect of WS when mixed with LSS as a representative case. For pure LSS and the 90% LSS-10% WS mixture (Figure 6a,b), Pb showed a higher tendency to form solid and liquid compounds (PbO) at lower temperatures. According to the TEC, as the temperature increased, Pb was predicted to convert into PbS (g) and elemental Pb (g). Starting at 600 °C, most of the Pb was calculated to shift to the gas phase, with only a small fraction remaining in liquid form at 700 °C. However, as more WS was added, the calculated liquid proportion of Pb in the biochar decreased further at 700 °C. The calculated behavior of Pb showed an increase in volatilization at elevated temperatures, particularly above 700 °C. As the proportion of WS increased, Pb volatilization was predicted to increase along with the formation of chlorinated Pb species. Pb is highly sensitive to temperature changes during pyrolysis, with a sharp increase in the gas phase starting at 600 °C and increasing with higher WS content. This suggests that the formation of Pb compounds is influenced not only by the temperature but also by the amounts of S and Cl present. Consistent with the MP-AES results shown in Figure 2, the calculated amount of Pb in the biochar from monopyrolysis is significantly higher compared to that from copyrolysis. As predicted by TEC, at 500 °C, most of the Pb remains in solid form (PbO (s)) and is retained in the biochar. However, with increasing temperature and the addition of WS, Pb forms volatile chlorides (PbCl (g), PbCl_2_ (g)) and sulfides (PbS (g)), resulting in its removal from the biochar (Figure 6b,c).

**Figure 6:**
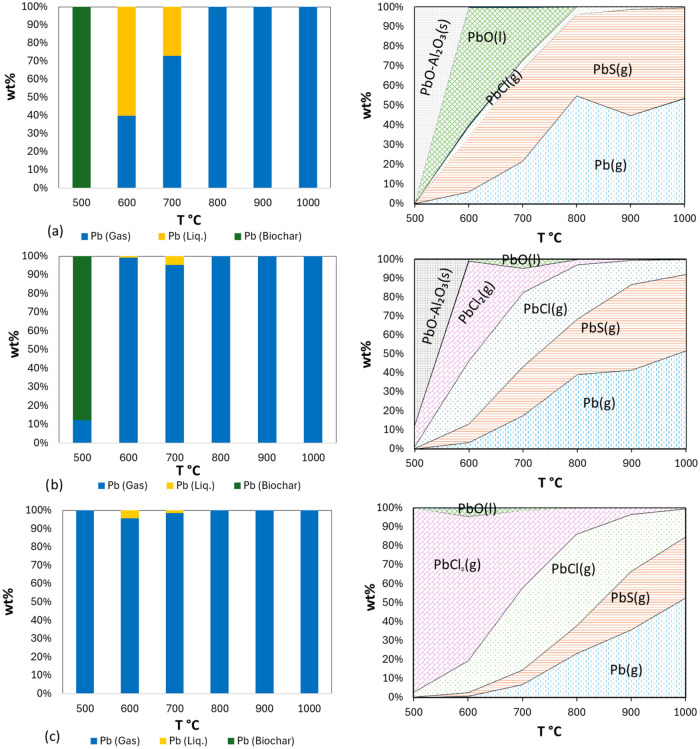
Predicted partitioning of Pb (left) and calculated Pb-containing compounds by TEC (right) during copyrolysis of LSS and WS at different temperatures, as wt % of Pb-containing compounds: (a) pure LSS, (b) 90% LSS-10% WS, (c) 70% LSS-30% WS.

Similar to Pb, Zn in the pure LSS (Figure 7a) exhibited minimal volatility below 700 °C, with over 90% of Zn retained in the biochar, according to calculations by TEC. At 700 °C, approximately 10–15 wt % of Zn was volatilized, increasing dramatically to nearly 100 wt % at 800 °C and beyond. When 10 wt % WS was introduced (Figure 7b), Zn volatility became more pronounced at 700 °C. By 800 °C, Zn retention in biochar dropped further, with the majority of Zn being released as Zn (g) and ZnCl_2_ (g). In the copyrolysis of 30 wt % WS with 70 wt % sludge (Figure 7c), Zn volatility increased even more, with 40 wt % of Zn separating from the biochar at 700 °C. This volatilization nearly doubled at 800 °C, where most of the Zn was found in the gas phase. The primary Zn compounds predicted during pyrolysis were ZnO, ZnS, and ZnFe_2_O_4,_ and small amounts of Zn_2_Si_2_O_6_, FeZnSi_2_O_6_ at lower temperatures. Above 700 °C, gaseous Zn, ZnCl_2_, and KZnCl_3_ became dominant, especially as the proportion of WS increased. Similar studies^80^ have shown that oxides of Al, Fe, and Si have an outstanding ability to capture HMs. Since WS contains a high concentration of Si, it is anticipated that adding WS to LSS in copyrolysis would enhance the enrichment and stability of HMs in the biochars. Therefore, increasing the proportion of WS promotes the volatilization of Zn, which begins releasing to the gas at lower temperatures 500 °C compared with monopyrolysis. This results in a higher proportion of Zn in the gaseous products through the chlorination reaction,^68^ followed by its capture in the flue gas cleaning system.

**Figure 7:**
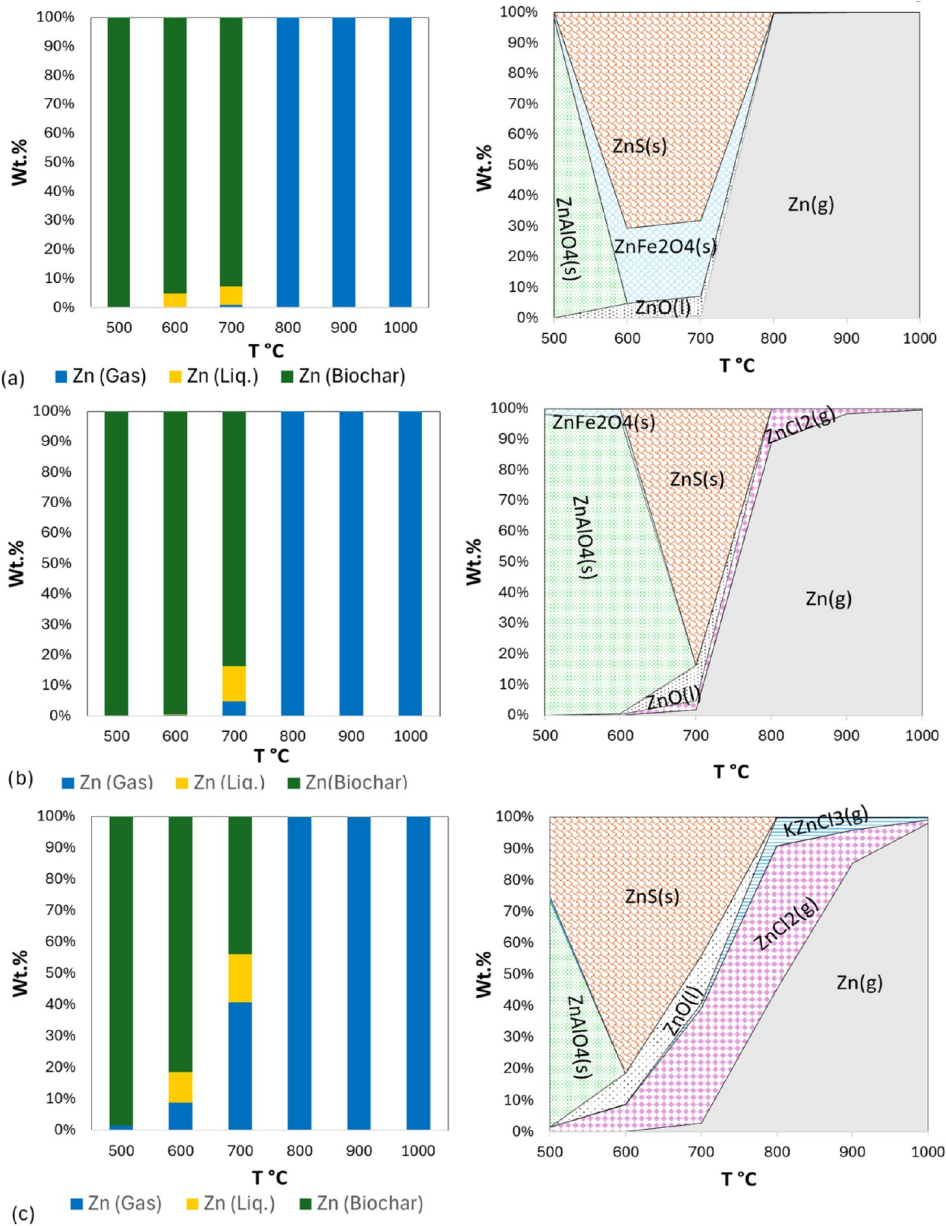
Predicted partitioning of Zn (left) and calculated Zn compounds by TEC (right) during copyrolysis of LSS and WS at different temperatures: (a) pure LSS, (b) 90% LSS-10% WS, and (c) 70% LSS-30% WS.

Regarding Cd, based on TEC, the volatility was significantly more influenced by temperature than by the addition of WS. At all studied temperatures and mixing ratios, Cd was predicted in gaseous forms such as Cd, Cd­(CH_3_), CdO, Cd­(OH), and Cd­(OH)_2_. However, Cu, Ni, and Cr were predominantly concentrated in the solid residue, which was consistent with the experimental results in Figure 2 in the form of Cu_5_FeS_4_, Cu_2_P_2_O_7_, Cu_2_O, CuS, and Fe_8_Cr_4_Ni_18_, respectively.

This study demonstrates that modeling with user-defined databases in HSC Chemistry and FactSage software effectively illustrates the influence of sulfur and chlorine on heavy metal immobilization, but it encounters certain restrictions. While the user-defined database offers more precise data for HMs, a significant problem arises from the elemental input technique, which often underestimates the significance of Si due to its intricate behavior and interactions with other elements during pyrolysis.^80,83^ In this study, the majority of Si was predicted to be in combination with alkaline earth metals (solution models from FactSage for feldspar and mullite), forming compounds such as K_2_CaSiO_4_, KAlSi_2_O_6_, KAlSi_3_O_8_, NaFeSi_3_O_8_, CaAl_2_Si_2_O_8_, and SiO_2_. Adding 30% WS increased the availability of alkali and alkaline earth metals, such as Ca, K, and Na. Si captured Fe and Al, which are abundant in MSS, and alkali and alkaline earth metals, such as Mg, Ca, Na, and K. This combination resulted in increasing the boiling point of KCl to 700 °C, thereby contributing to the retention of Zn at higher temperatures. As shown in Figure 5b, the clear diffraction peaks of SiO_2_, observed in the XRD diffraction patterns, indicate that Si plays a more significant role in HMs retention, particularly Zn, within the biochar.

To improve future TEC studies, a more detailed characterization of feedstocks, specifically focusing on Si compounds, such as silicates, should be integrated into the model. Furthermore, adjusting the atmospheric conditions and moisture content in the model to more closely mirror the reducing environment of pyrolysis could lead to more accurate results. Validation with experimental data is crucial for refining the model and ensuring that the thermodynamic predictions are in line with the actual process outcomes.

## Conclusions

This study demonstrates that both temperature and feedstock mixing ratio significantly affect yield distribution and the properties of biochar. Copyrolysis balances the yield of oil and gas, which helps reduce excessive volatile matter release while preserving favorable biochar characteristics. When agricultural residue feedstocks, such as wheat straw and bakery waste, are added during the copyrolysis of municipal sewage sludge, there is a notable reduction in some of the heavy metal concentrations in the final biochar. This reduction is attributed to dilution, as WS and BKW contain lower levels of these metals as well as potential synergistic interactions between the feedstocks. Such interactions can alter the chemical behavior of heavy metals, promoting their stabilization or facilitating their removal during pyrolysis. Consequently, copyrolysis with biomass not only reduces heavy metal content but also enhances the quality of biochar produced from MSS. While both WS and BKW improve biochar properties, WS has a more pronounced effect on surface area and pore structure. This makes it an effective feedstock for producing highly porous biochar suitable for environmental applications, such as soil conditioners. Increasing the amounts of WS in the feedstock promotes the formation of metal-stabilizing phosphates and calcium silicates, which aid in retaining heavy metals and P within the biochar. This effect becomes particularly significant at higher pyrolysis temperatures, where interactions between elements derived from WS (such as K and Ca) and components from sewage sludge promote the development of stable slag phases. For future simulations, it is recommended to include more detailed characterizations of feedstocks, specifically looking at Si compounds, such as silicates, which may play a crucial role during pyrolysis. Additionally, modifying the atmospheric conditions in the model to better represent a reducing environment would improve its accuracy. Incorporating the interactions between alkali and alkaline earth metals (such as K and Ca) with Si will also enhance our understanding of how heavy metals behave during the process. Continuous validation of the model against experimental data is essential for refining predictions and aligning them with actual process outcomes.
